# Co‐design of a complex school‐based intervention for attention deficit/hyperactivity disorder using the Intervention Mapping approach

**DOI:** 10.1002/jcv2.70169

**Published:** 2026-08-25

**Authors:** Abigail Russell, Suzie Holt, Ian Wellaway, Ali Hirst, Lynne Heaney, Rachel Hanna, James Wenger, Liz Hampton, Barry Sullivan, Fran Townsend, Tamsin Ford, Barnaby D. Dunn, Darren Moore, Edmund Sonuga‐Barke, Linda Pfiffner, Judi Kidger, Rachel Hayes, Rebecca Gudka, Eleanor Bryant, Kirsty Cordwell, the Tools for Schools Planning Group

**Affiliations:** ^1^ Children and Young People's Mental Health Research Collaboration, Department of Public Health and Sports Sciences University of Exeter Exeter UK; ^2^ Research IT University of Exeter Exeter UK; ^3^ Public Collaborator UK; ^4^ Department of Psychiatry University of Cambridge Cambridge UK; ^5^ Department of Psychology University of Exeter Exeter UK; ^6^ School of Education University of Exeter Exeter UK; ^7^ Institute of Psychiatry, Psychology and Neuroscience Kings College London London UK; ^8^ Department of Psychiatry University of California San Francisco San Francisco California USA; ^9^ Bristol Medical School University of Bristol Bristol UK

**Keywords:** ADHD, co‐design, digital health, Intervention Mapping, school‐based intervention

## Abstract

**Background:**

Attention deficit/hyperactivity disorder (ADHD) is a highly heritable neurodevelopmental condition, comprising varying difficulties in attention and/or impulsive and overly active behaviours, with knock on effects for health, learning, social and emotional functioning, self‐esteem and quality of life. There is little implementation of school‐based intervention for ADHD in UK mainstream primary schools, due to resource constraints and implementation challenges of existing, multi‐component complex interventions.

**Methods:**

This study describes the co‐design of a novel, personalised school‐based intervention for ADHD, delivered by teachers to primary school students. The Intervention Mapping approach was used to structure intervention development. Co‐design with a Planning Group of 33 adults and children with ADHD, school and health professionals is described in detail, as well as an overview of the resultant intervention: the Flex toolkit.

**Results:**

Co‐design was woven throughout the intervention development and evaluation process, leading to a novel digital health intervention for ADHD suitable for delivery in UK mainstream school settings, by classroom teachers. The Flex toolkit contains some core components applicable to every child, then becomes personalised depending on each child's areas of strength and challenge.

**Conclusion:**

This novel digital intervention builds on the existing evidence base whilst ensuring that the content, format and design as well as implementation and evaluation plan are suitable for the UK mainstream primary school context.

## Key Points

**What's Known**

- Existing school‐based complex interventions for attention deficit/hyperactivity disorder (ADHD) are not suitable for implementation in the UK school context, however the evidence base is extensive.
- Co‐design with members of the public is likely to improve the acceptability and potential for implementation of a novel school‐based intervention.

**What's New**

- In this study we report on the co‐design of a novel health intervention for ADHD designed to be delivered by classroom teachers in usual practice, based on existing evidence, and describe the resultant intervention.

**What's Relevant**

- The co‐design process and new intervention are relevant to health and education policymakers, educational practice and health service delivery in education settings, as well as to researchers developing behaviour change interventions.

## INTRODUCTION

Attention deficit/hyperactivity disorder (ADHD) is a highly heritable neurodevelopmental condition, comprising difficulties in attention and/or impulsive and over‐active behaviours (Faraone et al., 2021), with impacts on learning, socio‐emotional functioning, self‐esteem and quality of life (Shaw et al., 2012). It is one of the most common forms of neurodivergence, with a childhood prevalence of 2%–7% (Sayal et al., 2018). Classified as a disability under the UK Government (2010), ADHD typically presents in primary school (age 4–11 years) and often endures across adulthood. It is a highly variable condition with a 1:3 female:male ratio (Tarver et al., 2014).

Without treatment or support, ADHD results in significant impairment and co‐occurring health conditions with life‐long impacts on relationships, education, and employment (Shaw et al., 2012), and increased school exclusion, accidental injuries, and incarceration (Faraone et al., 2021). This places a considerable burden on‐ and is a major cost to‐society (Daley et al., 2019). However, as ADHD has been historically under‐recognised in UK settings, suitable training and resources for teachers are not available. Existing evidence‐based complex interventions are not fit for the UK context: they are often resource‐intensive and require specialised delivery (Pfiffner et al., 2016), however components that confer effectiveness could be synthesised and repackaged into a suitable solution (Moore et al., 2018; Staff et al., 2021). There is a pressing need for effective strategies that can be used within schools, able to cope with the diversity of need.

In UK mainstream primary schools the average class size is 26.1 students, with a single teacher present (Department for Education, 2026b). This teacher delivers all curriculum content, manages behaviour and safety, and completes school and government‐required data and monitoring activities for their class. 9.6% of teachers are currently leaving teaching in the state sector per year. Some schools will have teaching assistants (TAs) to support within‐class (an average of 11 per primary school) (Department for Education, 2026a) but TAs are often employed to support specific individual students 1:1 who have had funding provided to accommodate additional educational needs, to cover teacher absence or to withdraw small groups for curriculum‐based interventions. The teacher therefore usually manages the class alone, and has limited capacity to deliver personalised intervention work. Existing evidence‐based programmes such as the Collaborative Life Skills programme require facilitation of a child‐skills group, a teacher group and parent group (Pfiffner et al., 2016), and UK schools are operating at capacity and with a shortage of specialists such as Educational Psychologists, lacking adults with time to organise and deliver such small‐group interventions (Zuccollo et al., 2026).

Co‐design of a novel intervention may allow us to overcome implementation challenges whilst still drawing on the extensive relevant evidence base (Lyon et al., 2024). A Delphi study informed the outcomes that were considered of most relevance to people with ADHD, schools and families in terms of what a school‐based intervention should target (Perry et al., 2021). Whilst some of those that reached consensus had been well‐evidenced in primary school‐aged interventions, others had predominantly been researched in adolescent interventions (organisational skills), or not at all (self‐esteem). No single existing intervention existed that covered the breadth of these outcomes, so adaptation of an existing single intervention was not possible. In addition, initial patient and public involvement (PPI) in developing the research idea highlighted the cultural differences in how ADHD is understood between the USA (where most existing intervention studies have been conducted) that is dominated by a psychiatric model of understanding, and the UK where biopsychosocial understanding, developmental psychological and interface with the social model of disability are more dominant in how ADHD is conceptualised within schools.

Health‐based interventions situated within schools are described as ‘complex’ when they involve the cooperation of a number of individuals/groups; target multiple behaviours through components acting at different levels; and require flexibility in how components are delivered. The MRC/NIHR *Complex Intervention Research Framework* (2021) strongly underlines the need to include ‘diverse stakeholder perspectives’ at every stage of intervention development (Skivington et al., 2021). For ADHD, this requires an approach bringing together researchers, teaching staff, families, children, health and education specialists to tailor the intervention to the context and needs of children and schools. In this study the focus is on co‐design in the school setting. National Institute for Health Research (2021) describe *co‐design* as the ‘active participation of people with lived experience as important partners … throughout the design and delivery of services and support’. Such collaboration brings with it methodological challenge as researchers attempt to combine science‐based objectivity‐seeking with participatory paradigms (Hughes, 2003; E. J. S. Sonuga‐Barke et al., 2024) within a framework of institutional and funding constraints (Madden et al., 2020).

Co‐design in school‐based interventions is strongly advocated as it integrates theory, research, and practice to create scientifically robust, classroom‐relevant solutions. Combining classroom expertise with behaviour‐change design supports effective, sustainable interventions (Bevins & Price, 2014; Holmes et al., 2020; Hughes, 2003). Interventions that ignore complexity of school systems often fail at implementation; fit within school structures are critical (Moore et al., 2024) and incompatibility with school context is a common barrier to implementation (Long et al., 2016). Despite rising interest in school involvement in co‐design, its use appears to remain relatively niche and many studies highlight challenges. The literature is characterised by heterogeneous approaches, lack of detail on theories of change and co‐design experiences; and failure to evaluate effectiveness, reflecting practical challenges (Ey & Spears, 2020; Hughes, 2003; Reed et al., 2021). Broader factors such as entrenched professional/institutional practices that impair power sharing and difficulties recruiting representative PPI groups (Jesson & Spratt, 2017; Reed et al., 2021; Vella‐Brodrick et al., 2023) are apparent. Overall, despite claimed benefits, evidence for co‐design in schools remains sparse.

To co‐design a novel, personalised school‐based intervention for ADHD, the Intervention Mapping (IM) approach was used; a method for developing health promotion interventions based on principles of behaviour change (Eldredge et al., 2016). IM is well‐suited to this challenge as it is informed by the social‐ecological paradigm, highlighting interrelationships between individuals and environments. Taking a neuroaffirmative approach to ADHD (E. J. S. Sonuga‐Barke, 2023), the intervention considers how best to change the environment around the child, and determines whose behaviours need to change to support optimal outcomes. IM has six stages; co‐design, via a ‘Planning Group’ of PPI members, is integral to all of these (Eldredge et al., 2016). The neuroaffirmative approach is based in the social model of disability, whereby differences in brain development and functioning (neurodivergence) are conceptualised as part of normal human variation. Disability caused to neurodivergent people is a function of the world being set‐up for the ‘neurotypical majority’, which therefore results in impairment for the neurodivergent individual. This approach opens up explicit integration of individual (and group) strengths and affirmative approaches in intervention work, with more potential for environmental adaptations to improve outcomes for individuals, as opposed to viewing challenges as psychopathological and individually‐determined (E. Sonuga‐Barke & Thapar, 2021). Existing ADHD interventions have not taken this approach, presenting ADHD as something ‘within child’ that should be changed. Our novel approach instead places a focus on what can change *around* the child, to support reduction of impairment, whilst simultaneously recognising there are likely to be some individual inherent challenges relating to the neurobiological differences evidenced across those with ADHD (Faraone et al., 2021).

In this article, the process of co‐designing a behaviour change intervention to support children with impairing traits of ADHD in the UK Primary school context is described: the *Tools for Schools Flex toolkit* (henceforth *Flex*). Flex is a personalised, digital intervention that has several core components that teachers work through (psychoeducation, child strengths activity, functional behaviour assessment to operationalise two behavioural targets), that then feed into selection of two personalised strategies from six modules, with each module focussing on an outcome domain (e.g., hyperactivity). Strategies are a mix of individual and classroom or group‐based activities, delivered by teachers in routine practice. This study outlines our experience of applying co‐design principles at every stage of developing Flex, providing examples of the procedures undertaken; what went well; the benefits noted; and the challenges and constraints encountered. Study aims are to describe and reflect on the co‐design process, and to describe the resultant intervention.

## METHODS

### Design; Intervention Mapping and formation of co‐design Planning Group

IM is based on behaviour change; use of behaviour change techniques is associated with larger effects of teacher interventions to address social, emotional and behavioural needs (Merle et al., 2022). Co‐design of Flex and the resultant evaluation were chiefly informed by the PPI Planning Group (group henceforth ‘group’). Thie group is a core part of the IM process, comprised of key stakeholders and representatives of those who are to be end‐users or recipients of the intervention. They help to ensure that the project focuses on important issues for the community; that findings will be relevant locally; and to develop capacity for intervention and evaluation research (Eldredge et al., 2016). We describe this as PPI as the majority of those involved were members of the public, people with ADHD and families.

### Recruitment of Planning Group

Group members were recruited through a two‐stage process from 08/2020 to 02/2021. Flyers inviting people with relevant experience to an informal chat (Supporting Information S1: Appendix S1) were distributed via clinical networks, in the community and on social media. Versions were produced for reaching people with ADHD (of any age, particularly children, teenagers and young adults) and their families or carers, clinical experts, and school staff. Targeted recruitment of school staff and medical providers was also conducted by including these groups on general flyers advertising the Planning Group posted in community spaces, and sending specific tailored flyers to school networks and networks of clinical colleagues.

Interested individuals contacted A.R. to arrange an initial chat‐by phone or online. These informal 15–60 min interviews explored relevant experiences; shared project details; and sought input and feedback. A strengths‐based conceptualisation of ADHD was utilised throughout. This includes a neuroaffirmative approach as described above, alongside recognition that there are many aspects of children's lives that are successful or working well, which allows a focus on individual strengths and interests. Leveraging strengths is an aspect of positive psychology frameworks, that can help compensate for areas of weakness (Climie & Mastoras, 2015). This presents an opportunity to bring the intervention to the child in a manner that is positive and engaging, as opposed to being because there is something wrong with them that requires help. In ADHD a strengths‐based approach serves the mechanistic function of protecting/promoting self‐esteem, one of the toolkit outcomes. Strengths in ADHD are personal, and unlikely to be generalisable (although evidence on this is lacking), but may include creativity, thinking outside the box and being able to hyperfocus (Schippers et al., 2022). Identifying these upfront allows these to be harnessed in plans to overcome areas of challenge. Output from these interviews enabled PPI influence over the intervention's direction and development from the very earliest stages (summarised as ‘What works for ADHD’: Supporting Information S1: Appendix S2).

Following this, A.R. provided further information on co‐designing the intervention and research project, and gauged interest in joining the group. Those who expressed an interest were sent a document that laid out expectations and other details for example, confidentiality and reimbursement. A form collected information on consent for email addresses to be visible to group members (if not, they were blind‐copied into group emails); availability and duration preferences for group meetings; and an optional section to write a personal profile for the project website.

### Planning Group participants

Twenty three adults were recruited into the group (almost all of those initially spoken to), along with 10 children who provided input supported by their parent/carer. The group comprised: adults and children with ADHD, parents and carers of children with ADHD, teachers, TAs, educational psychologists, Special Educational Needs Co‐ordinators (SENCos), a GP, health psychologist, occupational therapist and a Child and Adolescent Mental Health Services registrar. 30% of adults were male, predominantly of White British ethnicity, and most children were male. Children who participated in the Planning Group ranged in age from 6 to 14 years old, and then older adolescents aged 19–25. Additional public engagement was conducted throughout, including with children, adding to core group members' ideas and direction across the development process (see section on ad hoc engagement, below).

### Planning Group meetings and intervention co‐design process

**FIGURE 1:**
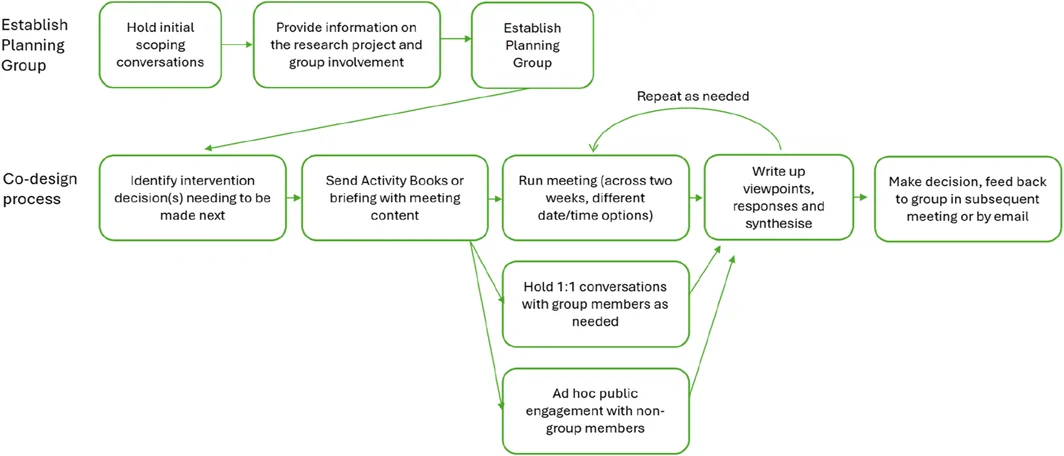
Overview of co‐design process.

A summary of the co‐design process is shown in Figure 1. Meetings were held online, due to the COVID‐19 pandemic. Whilst incurring some limitations, it enabled wider geographical involvement. Meeting times were scheduled to accommodate as many group members as possible. This usually meant running each meeting three times. As most people's preferences were for shorter meetings, meetings tended to be scheduled in two parts; part one at for example, Tuesday 7–8.30 PM, and part two the following week at the same time.

Group members were encouraged to contribute in varied ways to aid inclusion. Before meetings, ‘Activity Books’ were sent out to introduce topics, provide background information in lay language, and set out ADHD‐adaptive ‘chunked’ feedback spaces for members to record thoughts. The workbooks allowed non‐attendees to input and were used to scaffold in‐meeting discussion. Typically, meeting format comprised an update on the project stage, summary of previous meeting outcomes and resulting adaptations, followed by the tasks in the Activity Book and open discussion, facilitated by one researcher with a second taking notes. Discussion was allowed to flow freely. Ad hoc 1:1 meetings were offered for those not attending. Meetings usually contained input on Flex design/content, and training on the IM process. Additional drop‐in sessions were held when there were other areas to gather group input on, for example, when changes were made to the intervention, or fine‐grained examination of segments of behaviour change and associated intervention components. Other individual meetings were also held throughout, particularly when consulting on emerging findings, interpretation and implications of the feasibility study to ensure researcher decisions were grounded in group input.

Ad hoc engagement was also conducted to boost representation. Via schools of group SENCos, A.R. met with a range of parents of children with ADHD from different ethnic and sociodemographic backgrounds and varied ADHD profiles to hear their views, and with children to hear about what they enjoyed about school, and what they struggled with, along with ad hoc engagement with two further SENCos, four teachers and four TAs from multiple schools. Group members were reimbursed for their time in line with funder‐recommended rates per hour of contribution, with the choice of either monetary payment or vouchers.

### Analysis of Planning Group contributions

Detailed notes were written during and following group meetings, to capture input and ideas, with the same process being used to add further input from any written responses or ad hoc meetings. These were summarised by A.R. and brought to the group in the next meeting to discuss, explore how they aligned or differed from group views, and make decisions on how this additional input could be integrated. Decisions were made in the group meetings based on consensus, with A.R. or another facilitator keeping a written record of these to implement. The resulting outputs or changes were subsequently brought back to the group to review and refine. The stages of the co‐design process are described narratively, followed by a description of the resulting Flex development and intervention in Supporting Information S1: Appendix S3, reported in line with the ‘template for intervention description and replication’ checklist (Hoffmann et al., 2014). We did not conduct formal qualitative analysis for analysing feedback and engagement in co‐design, mainly due to time and resource constraints. Planning Group and ad hoc public contributors were not research participants and did not provide informed consent for their data to be recorded or analysed in a formal manner, and so we did not audio‐record meetings or capture data in a manner that would allow qualitative analysis. The subsequent evaluation study (Russell et al., 2026) did include a qualitative research component.

## RESULTS

### Planning Group input in co‐design

The group process was integral to the successful development of Flex. Stages of intervention development the group had input on are shown in Table 1. There was variation in points of view across the group for many of the decision points in the process. Where different views were expressed, these were discussed openly, sometimes immediately among the group and at other times A.R. would gather all opinions and bring back options to select from at the subsequent meeting. If there was one type of group member who was most proximal to the impacts of the decision, further views were collated from these representatives (e.g., if about home involvement in the toolkit, parent views were prioritised in decision‐making). Ultimately each decision was brought back as a recommendation to the group with a clear explanation for the reasoning for this, and consensus was reached on all occasions. This process was iterative, with some decisions being made quickly and others being discussed at multiple timepoints.

**TABLE 1:** Summary of Planning Group input into toolkit and research design.

1. Initial toolkit outline. This had to be developed prior to the funding application before formation of the group
2. Deciding toolkit outline, project name and logo
3. Drawing up a comprehensive list of behaviours seen in school that could map to each module in the toolkit (the ‘behaviour web’)
4. Brainstorming a range of strategies that could go into modules, based on lived experience of what works and does not work for different children with attention deficit/hyperactivity disorder
5. Toolkit design (the online platform and interface)
6. Design and conduct of the feasibility study in primary schools
7. Research ethics materials for the feasibility study
8. Reviewing toolkit outline, removing unnecessary components, adding new components
9. Reviewing and naming modules
10. Creating positive goals to match the behaviour web targets
11. Defining behaviour change steps (performance objectives)
12. Designing the wireframes for the toolkit (website layout drafts)
13. Designing the first prototype toolkit website
14. Alpha‐testing the prototype (first users of the prototype, testing out the features, navigation and interaction)
15. Deciding on changes to make to the toolkit during the research study, following feedback from schools
16. Considering implications of the feasibility study findings
17. Involvement in subsequent grant applications and publications

### Intervention description: The Flex toolkit

#### Co‐design in toolkit development

**FIGURE 2:**
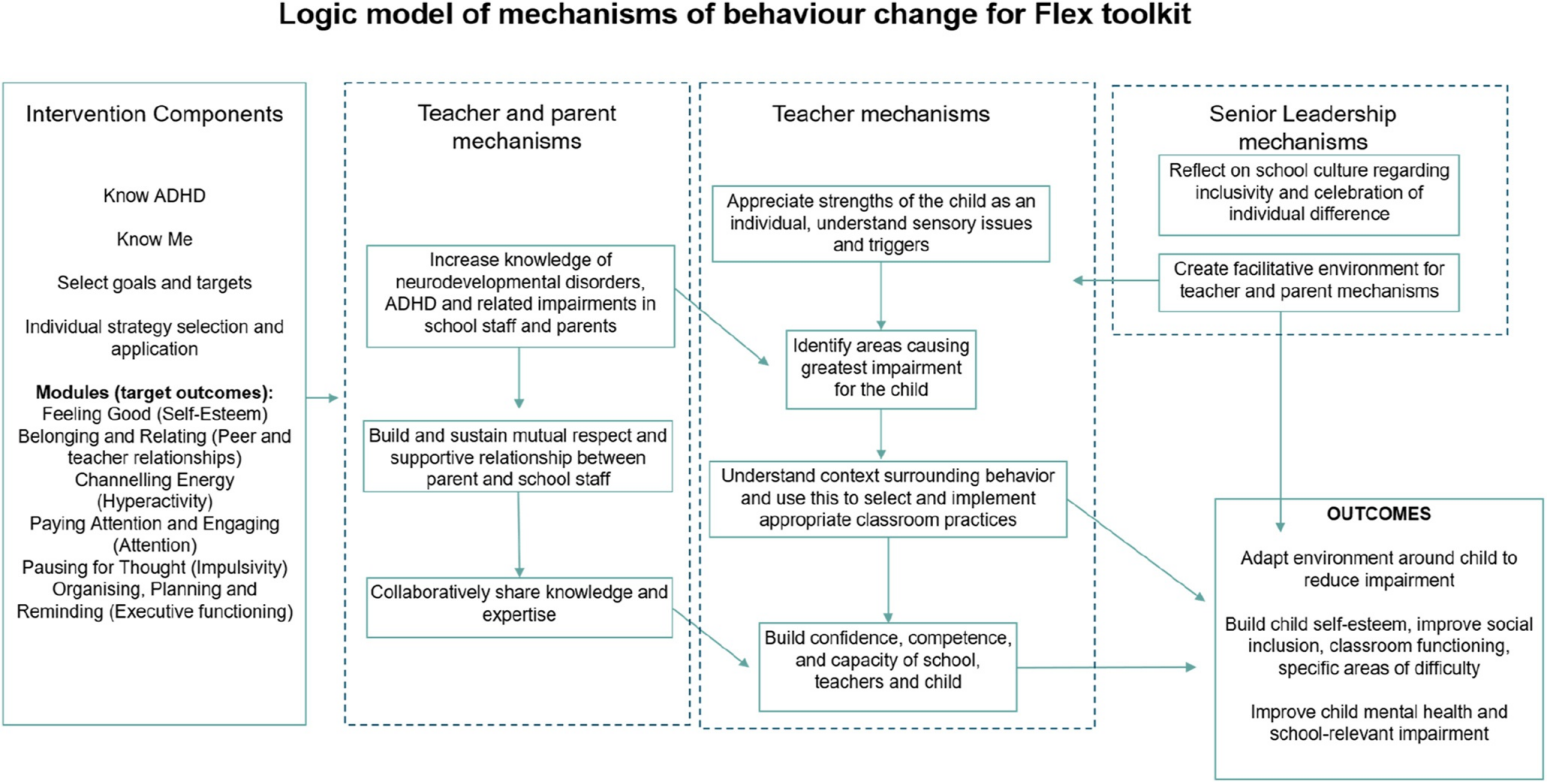
Flex logic model.

With group input in all the following stages, Flex was developed following an initial needs assessment and co‐development of logic models of the problem and solution, from which a logic model of change was constructed (Figure 2 and Supporting Information S2: Appendix S4). This was used to create a comprehensive list of the most basic behaviour changes needed by each agent in the system to reach the outcomes of interest, termed ‘performance objectives’ (henceforth ‘objectives’) in IM. These were initially divided into school staff, parent/carer, and child objectives (Supporting Information S1: Appendix S5). The comprehensive list was further distilled into a final list of objectives, with repetition removed. The objectives were then mapped by which behavioural determinant(s) (knowledge, attitudes, beliefs, values, self‐efficacy, expectations, skills, risk perception, anticipated regret, personal norm or subjective norm) would be relevant in changing each behaviour. The IM approach was followed for this, creating a matrix of performance objectives as rows and determinants as columns, working row‐by‐row to identify the relevant determinants for each objective (Eldredge et al., 2016).

Strategies and components of Flex were then designed in line with objectives, using behaviour change methods known to be of value, guided by the ‘change methods tables’ in Eldredge et al. (2016) that list evidence‐based methods for each determinant, along with parameters for effective use. Strategies and components combined a range of behaviour change methods, for example, for the objective ‘Teacher will assist child to recognise environmental factors that are triggers, and will communicate these with other staff and parents’ the strategy included methods of *consciousness‐raising, providing cues, shifting perspectives, implementation intentions, verbal persuasion (with a credible source of information), improving emotional states and goal setting*. With input from the group about what worked in their experience, and feedback on drafts from the group and research team, A.R. finalised strategies and components.

The final content for Flex was written by A.R. in collaboration with the group, with support from E.B., R.G., S.H. and K.C. to create resources. Wireframes and text were translated into a mobile‐compatible website format by I.W., working alongside A.R. A wireframe is a blueprint or visual outline of a website page, showing the layout and where different content would be placed on the page. The group tested the first ‘alpha version’ prototype of Flex, providing feedback on design, content and useability. The second ‘beta’ version was again tested by the group, with further refinements made prior to the feasibility study that aimed to assess acceptability, feasibility and perceived usefulness of Flex (Russell et al., 2026). The overall format is described in Supporting Information S1: Appendix S3; Supporting Information S1: Appendix S6 contains Flex screenshots.

#### Intervention name

Group members considered ADHD to be something that both should and could not be directly addressed by trying to ‘change the child’. For a child with ADHD to grow up with positive life experience and feeling happy with school, they would need their ADHD to be accepted and for adults to be flexible around their needs. The name for the toolkit, Flex, was proposed by A.R. following these discussions, endorsed by the group, as the central tenet of the toolkit was to ensure the school system was *flexible* around the child.

##### Initial toolkit outline

**FIGURE 3:**
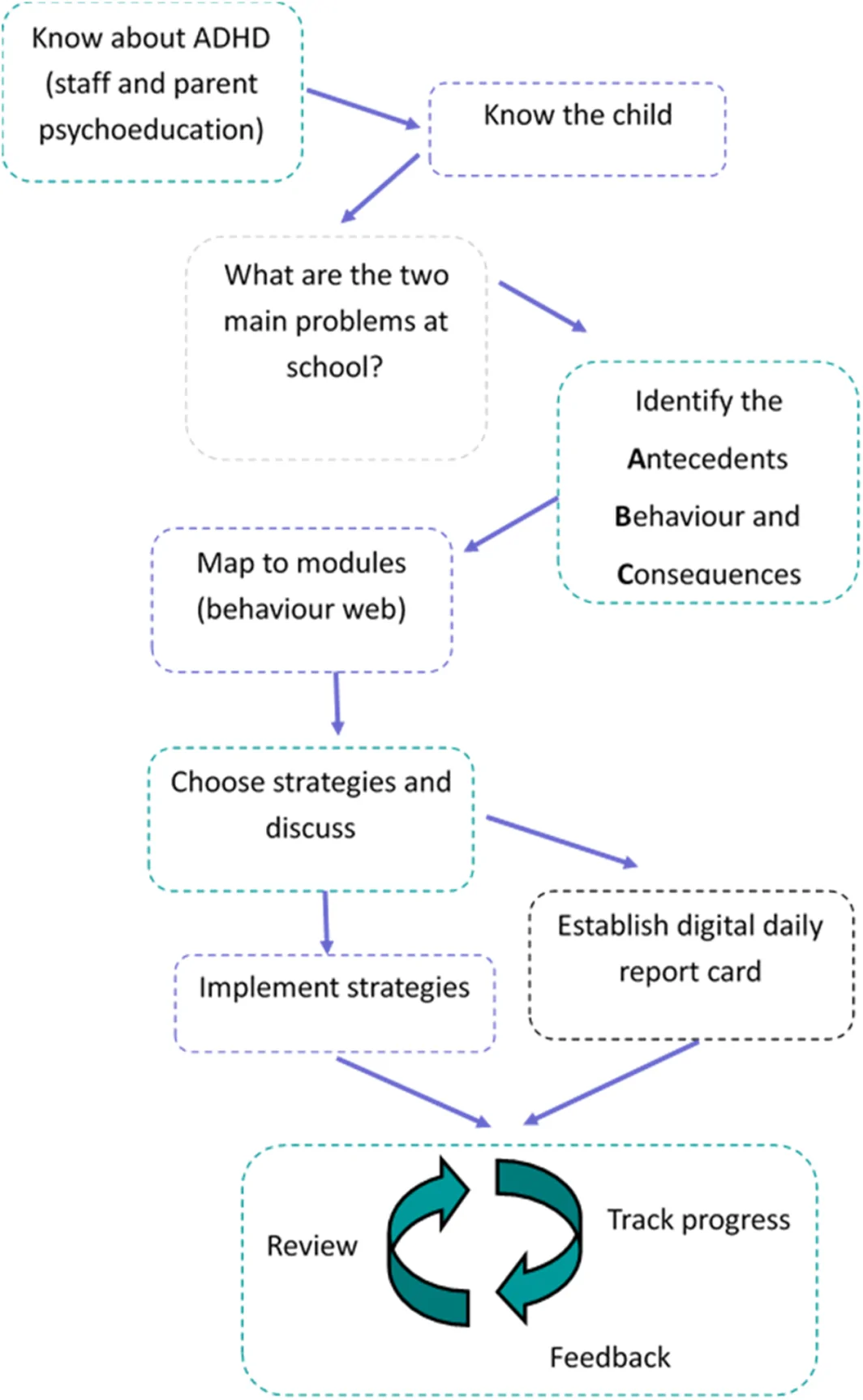
Flex toolkit process outline.

Group members collaborated on the final outline, refined from that developed with co‐design for the funding application (Figure 3). Detail on involvement of the child and home with Flex is in Supporting Information S1: Appendix S7.

#### Know about ADHD (staff and parent psychoeducation)

Group members considered it critical that the psychoeducation component was the same for both school staff and parents, to address any power imbalance that was often experienced. They also decided on topics the Know ADHD activity should cover, including sensory differences and scientific knowledge about ADHD, and the format of these: a series of short videos (totalling 45 min), hosted on YouTube. They also decided that this section should be titled ‘Know about attention, impulsivity and hyperactivity’ to avoid diagnostic language.

#### Know the child

The group felt that a large proportion of the problems seen in school in relation to ADHD could be mitigated by staff knowing the child better in terms of their strengths and interests and non‐verbal behavioural cues, because tailoring activities to a child's interests can support engagement. They highlighted that escalating negative situations at school could be prevented in many cases by adults recognising when a child was signalling dysregulation. The component was named ‘Know Me’ and was critical in highlighting the meaning of a child's behaviour, and indicators of when they were distressed, unhappy, or conversely indicated positive emotion or becoming over‐excited. Know Me was also designed to strengthen teacher‐student relationships: the group felt this was of critical importance. The format of Know Me activity was decided by the group to be flexible. Children with ADHD have different needs and interests, and whilst some would engage with a written activity, others prefer to make film clips, take photos or do crafts. Some had very specific ideas as to the best format, however consensus was obtained by including in the instructions a range of options that could then be personalised. The group considered it important for both home and school to complete Know Me, and share knowledge. Therefore, in Flex, home can see what the teacher has input, and vice versa. To make the child's voice prominent, the group suggested having two answers for each question: an answer from the child's point of view, and an answer from the adult.

#### What are the two main problems at school?

The group felt strongly that, in order to establish/improve relationships between school and home, there should be two meetings that laid the foundation for the targets the teacher would choose. This incorporated functional behavioural assessment (FBA): understanding what comes before and after a potential target behaviour, to understand what could be changed around the child to impact on the target. Templates of resources for school staff and home to conduct FBA were co‐developed alongside an exemplar, using non‐specialist terminology. The agenda for the meetings included reviewing child's strengths. Other group contributions included children's attendance being optional, the location of the meeting being flexible, with the option of families joining online from home. This was considered important in terms of addressing the power dynamic between school and home, and ensuring that, if parents/carers felt uncomfortable coming in to school they could join remotely. This component was rarely used by school staff in the feasibility study, as it was considered too time‐intensive and duplicative of usual goal‐setting processed. These findings were brought back to the group and refined to minimise the demand on school staff. This was met with disappointment by the group who felt the process was integral to a thorough understanding of a child's needs.

#### Map to modules (behaviour web)

The group were integral in forming the behaviour web, initially constructed using behaviours gathered in initial PPI chats in response to prompts around what behaviours relating to ADHD cause the most problems for a child in school. This was reviewed and refined, with some behaviours being merged, some dropped and others added, with each behaviour linked to relevant Flex modules. The behaviour web refining process was run as an activity in a group meeting, with a corresponding workbook activity. After reviewing the draft, group members were asked the following: ‘how can we represent things where two modules may be relevant, or should we collapse any two modules in to one?; what do you think of the behaviour web containing both positives and negatives?; what about the way it is designed?’ Written and verbal responses were listed and synthesised, with changes being made based on these responses. The functionality of this in the prototype was reviewed and approved by group members as being intuitive and helpful.

The key outcomes Flex was to target were determined in a Delphi study (Perry et al., 2021), and the group worked with the research team to decide how/whether to cluster these into modules. It was felt that more than six modules would be overly complex. Module names were proposed and selected by the group: rather than naming the negative attribute being targeted for example, hyperactivity, the names were positively phrased: for example, ‘Channelling energy’. Outcomes mapped on to the modules are shown in Table 2.

**TABLE 2:** Outcomes targeted and corresponding Flex toolkit module.

Outcome from Delphi study	Toolkit module
Inattention	Paying attention and engaging
Hyperactivity	Channelling energy
Impulsivity	Pausing for thought
Self‐esteem	Feeling good
Conflict with teachers and peers	Belonging and relating
Organisation skills	Organising, planning and reminding
Executive functioning	Organising, planning and reminding
Global functioning and quality of life	No specific module‐ all would contribute to these outcome depending on individual need

*Note*: Outcomes were identified from previous literature and/or proposed during the Delphi study (Perry et al., 2021) conducted to obtain consensus on the final list of outcomes the Flex toolkit should target.

When a teacher using Flex selected a module, they browsed the strategies within and selected one they thought would best suit the needs of the child, aligning with their classroom practice. This included a mix of individual and group‐level activities, as well as those that were used constantly in class, delivered as standalone activities, or implemented within teacher planning time to tailor delivery of learning within class. The group were divided as to whether individual or group activities would be best, recognising that individually‐applied strategies may not be implemented if a teacher did not have time, and feeling that group‐based strategies may be useful for improving consistency across the classroom. The final toolkit contained ∼1/4 individual, ∼1/2 group‐based, and ∼1/4 ‘teacher planning’ strategies, that the group were satisfied gave enough options to select something that felt feasible and useful for varied scenarios.

#### Strategy development process

Group input into the behavioural steps needed to produce positive outcomes based on the logic model of change were determined through a series of group meetings. The researchers listed the steps identified, and the group considered what behavioural determinants would need to change (e.g., skills, knowledge, awareness). These were mapped onto potential effective behaviour change methods by A.R., who drew together this information along with group input, theory around ADHD and learning, and existing evidence, and developed outlines for strategies to target outcomes. Strategy drafts were presented to the group, who added suggestions and refinements. Then instructions, in the form of ‘Plan’, ‘Do’ and ‘Review’ (commonly‐used terminology among teachers) and resources to support strategy implementation were created. Group members decided where one strategy was relevant to multiple modules. They considered that asking teachers to implement two strategies per school term with the student would lead to meaningful progress.

#### Establish digital daily report card

There is good evidence from research that daily, positive home‐school communication can be effective for children with ADHD (Iznardo et al., 2020). The group also felt that regular positive communication would be critical. As such, a notebook was built into the Flex prototype, although it was recognised by the group that schools may have other communication systems in place that they may prefer, rather than duplicating across multiple tools. The home‐school notebook was therefore optional, but school and home were asked to agree a schedule for positive communication and the format of this.

## DISCUSSION

This paper reports on the co‐design of Flex; a novel, individualised school‐based intervention for children age 4–11 with impairing traits of ADHD, including summarising the extent and impact of the co‐production process, and a description of the resulting intervention shown in Supporting Information S1: Appendix S3. IM provided a useful development structure that aligned well with input through co‐design, and was well‐received by the Planning Group. While logic modelling and intervention content development was led by the researchers, IM allowed for integration and exchange of group views and experiences at multiple points throughout, facilitated by the highly structured development process. Some stages of IM, such as the detailed evidence synthesis and production of logic models, required extremely fine‐grained detail that might risk making the process inaccessible for some members of the public. However, using summaries and splitting logic models into smaller components enabled group members to input into parts of the larger whole. We integrated group input throughout the whole logic modelling process, with researchers taking the wealth of information generated, distilling this and presenting streamlined models for review.

Planning Group input into Flex has been explicitly described in the results, illustrating how many of the stages and components were steered by lived experience. The sometimes‐differing views of parents/carers and school staff on occasion required careful management (e.g., to what extent home should be required to be involved), and when final decisions were described to the group and justified, they were met with unanimous support, even when the requests of some members could not be included. Families and school staff were integral to the group, as opposed to school staff being viewed as separate ‘expert stakeholders’. The integration of types of lived experience was carefully considered. Flex is a health‐based intervention designed for delivery by teachers in school settings. Not only did this require input from those with ADHD and their families, lived experience from both education and healthcare staff was critical to understanding the range of challenges, and what could work well to support health outcomes within a school setting. This meant the group included a diverse range of lay and professional views, that should enhance the acceptability and usefulness of Flex. Facilitation acknowledged the potential for power imbalances from the outset, and was careful to ensure that every group member was asked for their views, with contrasting views welcomed and explored within the wider group, and family and child voice foregrounded (Donetto et al., 2015; Oertzen et al., 2022). Part of the success of this was the initial chats with group members and other people with ADHD (Supporting Information S1: Appendix S2), with the initial reference document clearly outlining ADHD from a child and person‐with‐ADHD perspective, prior to the involvement of health and education professions.

Whilst group input was evident across the process of obtaining funding, planning and developing the intervention, it was not embedded throughout *every* stage of the research process. Some decisions were constrained by the funding/resources available, and there was limited capacity to revisit decisions already made. Holmes et al. (2020) formalised this in their study by making explicit distinction between ‘modifiable’ and ‘non‐modifiable’ elements of intervention and design. This explicit distinction was not made in the current study, and there was the possibility of complete re‐design should that prove necessary. Constraints on input were particularly a challenge for those in the group who joined the project in the later stages. A strength however was the diversity of views and experiences represented, which was bolstered by the flexibility to involve new group members as the study progressed. A summary of recommendations for others co‐designing behaviour change interventions is included in Supporting Information S1: Appendix S8.

Flex is novel in design and delivery, building directly on existing evidence to reduce research waste and avoid duplication of effort (Bleijenberg et al., 2018; Moher et al., 2016). The novelty of Flex lies within the packaging of a wide range of evidence‐based components as stand‐alone strategies or activities, with personalised selection depending on the needs of the child. This has not previously been reported in school‐based ADHD interventions, which instead deliver entire multi‐component programmes as entire units to all participating children. Further novelty is the teacher‐delivered mode of the intervention, not requiring a specialist to apply within the classroom, and the mixture of individual and group strategies, meaning that the intervention can be tailored to integration in routine classroom practice, or used 1:1 with a child. While there is substantial evidence regarding non‐pharmacological and school‐based ADHD interventions (Moore et al., 2019), few are suitable for implementation in the UK school context and resource‐constrained school systems, for example, involving dedicated within‐ or after‐school sessions for small groups staffed by a clinician or social worker and requiring active school‐home collaboration (Pfiffner et al., 2016). Co‐design was considered integral to understanding the organisational constraints and facilitators in the contemporary primary education system in the UK in delivering a health‐based intervention (Ramos et al., 2020). Enabling non‐specialists to deliver a personalised intervention to a heterogeneous range of children, Flex aims to streamline the resources needed for delivery and overcome implementation barriers; this was tested in the subsequent feasibility study (Russell et al., 2026). Evidence was also incorporated from research fields beyond ADHD relating to outcomes of interest. This maximises the chances that there will be a suitable strategy for every child within the remit of Flex.

To ensure Flex can be personalised, and given the range of evidence available, group input helped to ensure that it includes many strategies beyond its core components. This tailored/personalised approach is being increasingly tested, meaning that one intervention could have benefits for a transdiagnostic population (Black et al., 2018, 2023); a recent systematic review identified 13 studies comparing digital health interventions with personalised and non‐personalised components, though finding little evidence of increased benefit at this stage (Schaeuffele et al., 2025). Working with a wide range of group members helped to generate logic models and resultant components that spoke to the heterogeneity of ADHD and therefore a range of potential strategies that could be selected depending on the individual student‐classroom fit. Qualitative research with parents about conduct problem interventions highlights the need and benefits of personalisation (McKay et al., 2020), however robust evidence of whether this enhances effectiveness is needed. A multi‐component intervention such as Flex could also be evaluated for effectiveness in a series of micro‐trials of individual components, building on recent work in parent and teacher ADHD interventions (Staff et al., 2021, 2022).

Thinking ahead to widespread implementation, there are further advantages in the personalised approach relevant to the school‐level. A recent meta‐analysis with a focus on interventions being delivered across multiple contexts reported that interventions adapted for each context were the most effective, relative to novel and adopted interventions (Olsson et al., 2024). As such, embedding points where Flex can be adapted across schools as well as to individual children leads to a greater potential for effectiveness. Co‐design also brings benefits to future stages of the research process; evidence suggests that ‘PPI interventions’ in trials are associated with increased participant enrolment (Crocker et al., 2018).

### Strengths and limitations

Co‐design between researchers, educational professionals and families was fundamental in developing the Flex toolkit and was a constant thread running throughout this project. At times, this collaboration achieved full ‘co‐production’, for example, in developing the logic models, whilst at other times inherent barriers limited our attempts to achieve this, such as exhaustively listing the performance objectives required to produce behaviour change. Capturing data on exactly what decisions were attributable to co‐design was beyond the remit of the project. As such, it may be that group members have a different opinion to the research team as to the depth and success of this process, although seven are individual co‐authors on this paper, endorsing convergence of views. Future studies could utilise systems such as the ‘Public involvement in research impact toolkit’ Newman et al. (2023) to capture decision points throughout the process and obtain input into whether there is agreement that these were indeed co‐produced (Mann et al., 2018).

Limitations of online meetings were that conversation did not flow as naturally as it would if a group of individuals met in person. In natural dialogue, people often talk over one‐another, or express agreement or disagreement in tandem and this was more challenging with online meetings as group members politely listened for others to express their views. There could then be points of hesitation where multiple members had thoughts to share but did not want to speak over someone else. This was overcome by careful facilitation, ensuring that all views were sought (e.g., asking individuals by name what they thought about a point that had been made) and asking for clarification where it looked like there was consensus (e.g., ‘I can see you were all nodding when [name] spoke. Does anyone have a different view on this?’).

There is also the potential for bias in the development process as a result of who engaged with the group, or potential to miss other varied viewpoints. Those parents/carers keen to be involved in a group and research are engaged and often involved heavily in their child's support for their ADHD, which will not be representative of all parent/carers whose child may receive the intervention. The group may represent a ‘best case scenario’ of engagement. Factoring in consideration of intervention users with varied engagement as well as consideration of factors such as teachers with varying levels of experience is critical. Including ad hoc engagement facilitated through schools has enabled some explicit consideration of these, and gathering of varied opinions throughout the process should result in a more robust delivery model and expectations. Feasibility‐testing of the toolkit has been completed at the time of finalising this paper (Russell et al., 2026), and so next steps are to integrate study findings and consider adaptations to Flex based on feasibility findings before proceeding to further evaluation (Reed et al., 2021).

## CONCLUSIONS

This study reports the co‐design of a school‐based behaviour change intervention for ADHD grounded in the lived experience of intervention users and recipients. This novel digital intervention builds on the existing evidence base whilst ensuring that the content, format and design as well as implementation and evaluation plan are suitable for the UK mainstream primary school context.

## AUTHOR CONTRIBUTIONS

**Abigail Russell**: Conceptualization; investigation; funding acquisition; writing—original draft; methodology; writing—review and editing; formal analysis; supervision; resources; project administration; data curation. **Suzie Holt**: Investigation; writing—original draft; writing—review and editing; formal analysis; methodology; project administration. **Ian Wellaway**: Methodology; writing—review and editing; software. **Ali Hirst**: Writing—review and editing; methodology; formal analysis. **Lynne Heaney**: Methodology; writing—review and editing; formal analysis. **Rachel Hanna**: Methodology; writing—review and editing; formal analysis. **James Wenger**: Formal analysis; methodology; writing—review and editing. **Liz Hampton**: Methodology; writing—review and editing; formal analysis. **Barry Sullivan**: Methodology; writing—review and editing; formal analysis. **Fran Townsend**: Methodology; writing—review and editing; formal analysis. **Tamsin Ford**: Conceptualization; writing—review and editing; funding acquisition; methodology. **Barnaby D. Dunn**: Conceptualization; writing—review and editing; funding acquisition; methodology. **Darren Moore**: Conceptualization; writing—review and editing; methodology. **Edmund Sonuga‐Barke**: Conceptualization; methodology; writing—review and editing. **Linda Pfiffner**: Conceptualization; methodology; writing—review and editing. **Judi Kidger**: Conceptualization; methodology; writing—review and editing. **Rachel Hayes**: Conceptualization; methodology; writing—review and editing. **Rebecca Gudka**: Methodology; writing—review and editing. **Eleanor Bryant**: Methodology; writing—review and editing. **Kirsty Cordwell**: Methodology; writing—review and editing. **The Tools for Schools Planning Group**: Conceptualization; investigation; methodology; funding acquisition; writing—review and editing.

## CONFLICT OF INTEREST STATEMENT

The authors declare no conflicts of interest.

## ETHICAL CONSIDERATIONS

Ethical approval was provided by the University of Exeter College of Medicine and Health Research Ethics Committee (Jan22/B/300); approval obtained 24 January 2022. Informed consent has been appropriately obtained from research participants, with informal verbal consent and a signed confidentiality agreement from Planning Group (PPI members). This article contains no primary research data requiring patient consent.

## AI STATEMENT

The Authors declare that they have not used AI in any capacity that would require disclosure in accordance with Wiley's AI Guidelines, nor in any capacity that would reasonably require discloser for editors, reviewers and readers to properly evaluate their research or manuscript. The authors confirm that AI has not been used for purposes including but NOT limited to drafting and editing, to generate substantial text or restructure arguments, nor in the research methodology. The authors take full responsibility for the accuracy of this statement.

## Supporting information

Supporting Information S1

Supporting Information S2

## Data Availability

The data that support the findings of this study are available on request from the corresponding author. The data are not publicly available due to privacy or ethical restrictions.
