# Supplementary material for: Co‐design of a complex school‐based intervention for attention deficit/hyperactivity disorder using the Intervention Mapping approach
Source: JCPP Adv. 2026 Aug 25:e70169. Online ahead of print. doi: 10.1002/jcv2.70169 (PMC13507638; doi:10.1002/jcv2.70169)
Supplement: Supplementary file 1 [file JCV2-9999-e70169-s001.docx]

# **Co-design of a complex school-based intervention for attention deficit/hyperactivity disorder (ADHD) using the Intervention Mapping Approach**

# **Supporting Information**

## Appendix S1. Flyer used to recruit the study Planning Group and project overview flyer

## Appendix S2. “What works for ADHD”- write up of initial involvement and engagement chats

# What works supporting a child with ADHD? Findings from informal chats with people with relevant experience regarding ADHD and Primary School

Abby Russell – February 2021. Version 2 - long form

## Introduction and background

This document discusses findings from informal conversations with people about ADHD conducted as patient and public involvement for the Tools for Schools project. The aim was to understand the current context of being a child with ADHD in a UK Primary school, and understand what works, and why these things might work, to help support children with ADHD.

I approached and spoke to people with ADHD (adults and children), parents and families of people with ADHD, a range of school staff and special educational needs co-ordinators (SENCos), psychologists (including educational and clinical psychologists), psychiatrists, occupational therapists and ADHD nurses. Notes from phone calls, presentation discussions and team meetings have been synthesised together using a narrative approach (looking for common themes to group things together) and then been written up by me. Please be aware that these are reflections of personal experiences and are not research data. They are not evidence-based in that I have not assessed whether there is scientific evidence about these topics, but they represent people’s lived experience of the topic. Any names mentioned in this document are pseudonyms (i.e. not people’s real name).

Table of Contents

[1](#_Toc140218648)

[Introduction and background	1](#_Toc140218650)

[Things to know about children with ADHD	3](#_Toc140218651)

[What is ADHD? What does it look like?	3](#_Toc140218652)

[Who is involved with ADHD in Primary school?	5](#_Toc140218653)

[Understanding why children with ADHD behave ‘badly’ at school	6](#_Toc140218654)

[Change your expectations?	6](#_Toc140218655)

[Understanding the diagnosis process	6](#_Toc140218656)

[Supporting parents/families	7](#_Toc140218657)

[Enjoyment=engagement	7](#_Toc140218658)

[Supporting school staff	7](#_Toc140218659)

[Things that some children with ADHD like:	8](#_Toc140218660)

[Things that don’t suit some children with ADHD:	8](#_Toc140218661)

[Friends	8](#_Toc140218662)

[Learning	8](#_Toc140218663)

[Teaching a new skill	9](#_Toc140218664)

[How do you know what a child with ADHD does or doesn’t like?	9](#_Toc140218665)

[Visual tools	9](#_Toc140218666)

[Short, clear instructions and ‘chunks’	10](#_Toc140218667)

[Lists	10](#_Toc140218668)

[Balance of adult involvement and supervision	10](#_Toc140218669)

[Unstructured times	10](#_Toc140218670)

[Written work	11](#_Toc140218671)

[Focus	11](#_Toc140218672)

[Self-esteem and success (exclusion and isolation)	11](#_Toc140218673)

[Set them up to achieve	12](#_Toc140218674)

[Other parents and children	12](#_Toc140218675)

[Praise	12](#_Toc140218676)

[Flexible expectations	13](#_Toc140218677)

[Reduced and flexible timetables	13](#_Toc140218678)

[Group work	14](#_Toc140218679)

[Communication and Tensions between school and home	14](#_Toc140218680)

[School-level policies	15](#_Toc140218681)

[Fitting in to school	15](#_Toc140218682)

[Less waiting	16](#_Toc140218683)

[Do things gradually	16](#_Toc140218684)

[Active listening and building empathy	16](#_Toc140218685)

[Support self-regulation	16](#_Toc140218686)

[How do you get to know a child with ADHD?	17](#_Toc140218687)

[Persistence	17](#_Toc140218688)

[Reflecting on past situations	17](#_Toc140218689)

[Keeping occupied	17](#_Toc140218690)

[Other ADHD treatments (medication)	18](#_Toc140218691)

[Want to share this document or know more?	18](#_Toc140218692)

## Things to know about children with ADHD

Children with ADHD do not intentionally behave in disruptive ways the majority of the time, however as they mature they become very aware of negative reactions to them and their behaviour, internalising criticism which could exacerbate low self-esteem. There are many reasons that the core symptoms of ADHD may be shown, these include a child not being able to control their urge to fidget and move about and therefore unintentionally not staying in their seat in a lesson.

ADHD can mean that a child struggles with academic tasks, like writing and reading, studying quietly on their own for long periods of time, and completing work.

Children with ADHD often want to be seen as ‘normal’ and feel like they fit in, as we all do. Finding ways to make sure they feel part of the school and class is important (lots of ideas on how to do this below).

## What is ADHD? What does it look like?

The following are examples people have given of how their ADHD affects them:

1. Work takes hours because of struggling to concentrate
2. Poor attention
3. Not very good with deadlines
4. Attention is better in the morning
5. Can only focus if interested
6. Rarely ‘just’ ADHD (often lots of problems combining together)
7. Not very good at routines, although they try hard
8. No time awareness
9. Untidy uniform
10. Exercise to concentrate more and fidget less
11. Handwriting and typing laborious
12. Lacking common sense
13. Drifting off
14. Not able to verbalise feelings and thoughts
15. Talking in school
16. Fixed way of thinking
17. Struggle with productivity
18. Not finishing work in class
19. Talking back to teachers
20. Others lead them into trouble
21. Quiet, unnoticed
22. In the wrong place a lot of the time
23. Not in the right place a lot of the time
24. Forget to bring things home from school
25. Point out when people do things wrong (little social grace)
26. Petrified of failing if they are good at something
27. Information-processing difficulties
28. Discrepancy between processing speed and intelligence
29. Emotionally intelligent
30. Profound and articulate
31. Logical
32. Practical
33. Bright
34. Quirky
35. Hyper-sensitive
36. Hypo-sensitive
37. Difficult to sit still and listen quietly
38. Difficult to engage with boring subject
39. Defiant or argumentative
40. Not knowing the moral rules (stealing things)
41. Fights
42. Problems with buttons and zips and fine motor skills
43. Skipping school
44. Running away
45. Climbing high things
46. Getting into trouble
47. Hyper
48. Fast metabolism
49. Can’t concentrate to have a coherent conversation
50. Rollercoaster
51. Can’t maintain friendships but can make friends
52. Anxious
53. Excessive talking
54. Speed reading
55. Sleep problems
56. Can’t stay in a seat
57. Inattentive
58. Prefer adult conversation and company

## Who is involved with ADHD in Primary school?

This graphic illustrates the most commonly occurring networks. Thicker arrows represent more important relationships. Dotted arrows represent potential relationships but less spoken-about

## Understanding why children with ADHD behave ‘badly’ at school

Children with ADHD may be very sensitive to noise and other stimulation, similar to children with Autism spectrum conditions. Therefore someone raising their voice to the child may provoke an extreme reaction or lead to behaviour that is disruptive or unacceptable. There are several examples where children with ADHD have reported intensely negative feelings towards teachers who have intimidated them, embarrassed them, or who have not taken the time to listen to the child’s perspective. People with ADHD who are very sensitive to stimulation may therefore find teachers and school staff to be scary if they raised their voice, shout or are overly energetic.

When children with ADHD do have an outburst of disruptive behaviour this is often beyond their conscious control and related to feelings they cannot express verbally or calmly. Once they have calmed down and reflected they are likely to feel remorse for their reaction, and may feel ashamed by their behaviour. When a child is aware of their ADHD diagnosis, they may assume that others would know that they are not in control of how they behave and feel like injustice has been done if this is not recognised. They may feel unduly punished.

Understanding the reason for challenging behaviour will help understand how to support the young person. Some thought these were mostly driven by either *attitude* or *anxiety*, and thought sensory issues could play a role in explaining and understanding the problem.

## Change your expectations?

Several parents mentioned that they had changed their expectations for what they want for their child, often through the process of diagnosis and learning more about ADHD. It seems sadly common that children with ADHD do not reach the same academic potential as other children with equal intellectual ability, and their ADHD symptoms mean that even if they are desperate to learn, they may not achieve as highly as their peers. Several families said they now just want their child to be happy and fulfilled. Teachers could consider adjusting their expectations in a similar way, praising the child for small successes and being aware of the disparity between someone’s ‘potential’ and actual achievement.

## Understanding the diagnosis process

People perceived that clinical services for ADHD were over-burdened, and can feel unsupported by these services, especially if they do not choose to have medication. If there is an EHCP in place it needs to be considered by the LEA and the school to see what accommodations could help support the child. Parents often said that they noticed that their child was ‘not like other children’ when they were relatively young, and may need the school to support them during the diagnosis process. Schools have varying involvement in this process and it was felt that different schools may have very different opinions of the same child depending on their ethos. Psychologists and clinicians reported that they usually contact a child’s school during the diagnosis process, requesting that the teacher fills in a behaviour questionnaire, provides a behaviour log for the past 6-12 months, and the child’s school report. On occasion clinicians would speak directly to the teacher or conduct an observation of the child while they were at school.

People said that overall a lot of different services could end up involved in assessing or supporting a child’s needs.

## Supporting parents/families

Parents find the process of referral and diagnosis, as well as navigating between different services, difficult and confusing. This is not helped by receiving different information from different professionals.

Peoples’ understanding of ADHD may be influenced by the media and their culture, this could affect whether they are accepting and understanding of an individual child. Schools may attribute traits of ADHD to problems with the home environment or parents, however ADHD has a strong genetic component and it is likely that parents of children with ADHD have some of these traits themselves. School staff could consider the challenges a family may be facing in relation to their interactions with the school, and show empathy. For example, parents may have low literacy levels, ADHD, and had their own negative experiences of school that affect their attitude towards their child’s school.

Family and parent support groups were seen as a safe space for parents, and exist across the UK. Some people felt these were very helpful, especially in terms of sharing ideas on how best to support and manage ADHD. Parents felt they benefitted from support around diet, exercise and sleep hygiene advice. Parent and family groups sometimes also involve activities for children, and may be in person or on social media platforms.

Social media can be a very negative experience for parents of children with ADHD.

## Enjoyment=engagement

What does the child engage with and enjoy? Engage by tapping into interests.

## Supporting school staff

People felt that school staff in general needed a lot more knowledge (training) and support to effectively work with a child with ADHD. SENCos and educational psychologists were perceived generally to be best-equipped to provide advice and guidance, although schools would appreciate more input from clinical services such as CAMHS or paediatrics. Educational psychologists are often asked for resources by school staff.

Specific things that would help to support school staff included training all staff in inclusion and awareness of ADHD, including mealtime assistants and teaching assistants. People often felt that although these staff were often well-intentioned, their lack of formal training meant they were not well equipped to manage potential trigger situations that could snowball into challenging behaviour and even result in exclusion. It was noted that often the staff overseeing unstructured times at lunch and breaks were mealtime support or teaching assistants, and so training them is important. It was felt that TAs should receive training on ADHD and autism, especially as their role often involves supporting these children in the classroom.

It was felt that there could be better communication about Education Health and Care Plans. People felt that teachers and TAs needed to be better informed about the content of plans, and the actions they needed to take to implement this.

Some people pointed out that teachers often face difficult conversations with parents of children with ADHD, and sometimes they are not well equipped for this. Parents acknowledge that there is need for these conversations so that they can understand what is happening at school. Providing teachers with the tools to have difficult conversations and appropriate supervision and support was thought to be important.

## Things that some children with ADHD like:

Outdoors, forest school, dog walking, building dens, art, drawing, climbing, Minecraft, fishing, tractors, Lego, building/constructing things, facts, trivia, obscure facts, The Simpsons, gymnastics, dance, sports. Things to keep busy during unstructured times: dot-to-dot, cut-outs, varied puzzle books. Children like to participate in clubs and activities, but seem to have a more positive experience when these are individual activities within a club (e.g. climbing).

## Things that don’t suit some children with ADHD:

Team sports (like football, rugby), being isolated or singled out, writing.

## Friends

Children with ADHD may struggle to make or maintain friends, again this varies a lot for each child. Some children with ADHD get on very well in 1:1 situations with their peers, although they may need an adult to structure the play or supervise from a distance. For example, when a friend comes round for an hour, they could play with Lego for 15 minutes, go outside and ride bikes for 15 minutes, have a 15 minute snack and then play computer for the remaining time. Team sports seem to be problematic for the most part for these children, as they can struggle to read social cues and managing relationships with multiple other children as well as playing a game like football and adhering to the rules of the game might be overwhelming.

Children with ADHD say they like having friends to talk to, and enjoyed having an identity with that group, for example someone mentioned they were the group of ‘weird friends’. They look up to the ‘cool kids’ in the classroom and school as role models, and one person jokingly said they would have worked harder in school if they had sat next to the prettiest girl in class.

## Learning

Topic-based learning that catches the child’s interests is important. Periods of learning that involve physical activity, such as active maths lessons, moving to different corners of the room to reflect an answer to a question, using colourful balls to represent things are ways to engage the child’s interests and therefore are more likely to try to pay attention. This active learning is helpful because it involves physical movement, a positive source of distraction (or engagement) in the learning materials which are nice to play with.

Tailoring learning activities to the child’s interests may be the only way to get them to engage with a subject that they do not like or find difficult (although this may also be dependent on pupil-staff relationships for these subjects).

How can we catch a child’s interests? Relate learning to things they already know about or are keen on, for example one family does home science experiments relating to their family pets, another encourage their child to read by having them read to the family dog. Use metaphors and analogies that link to what they like, for example if a child is very interested in cars, an analogy where you discuss whether a car would pass or fail it’s MOT may help to engage them in discussion on another topic.

## Teaching a new skill

People have said that the best ways they’ve found to learn new skills involve an adult showing the child how a strategy works by physically performing the activities the child would be expected to do (known as “modelling”), or role-playing what an appropriate response would be. The child should then be supported to practise this skill with the adult until they can do it independently themselves.

## How do you know what a child with ADHD does or doesn’t like?

Like all children, children with ADHD are very different from one-another. Some children with ADHD will have good verbal communication skills and be able to explain what they like, what they find aversive (or don’t like), and the reasons for this. However other children may be unable to express their thoughts and feelings verbally in the way we might expect of other children the same age.

It is very important to understand how an individual child with ADHD expresses their feelings, and how they behave when they are happy or sad, excited or anxious. Knowing the child well is important, as they may become excitable and agitated when they enjoy something, in a different way to being excitable and agitated when they are worried about something. Learning to read these signals will help school staff to understand when situations might be escalating out of control. Finding out what a child really likes (e.g. The Simpsons) and directly observing how they behave when they are enjoying something (e.g. doing an activity to draw your favourite Simpsons character) can help to understand the range of their behaviour, and to prevent misinterpretation of potentially-positive situations.

Visual tools are therefore important to help children with ADHD to communicate with adults and to begin to self-reflect on their emotional state or other important regulatory factors. These can be used to help with time management, for example using a timer or a clock that the child can clearly see, to help them keep track of tasks, with a visual representation of what tasks they are doing now, and what is next. Others use systems such as ‘traffic light’ colour indicators for children to indicate their mood, or ‘sensory ladders’ to capture different states of arousal and emotion on a visual scale.

Visual reminders can also be used to help a child remember the rules they are supposed to abide by in a given lesson, for example ‘blurt blocks’ where every child starts with five blocks on their desk, and they lose a block should they shout out in class.

## Short, clear instructions and ‘chunks’

One thing that children with ADHD struggle with is keeping track of what they are supposed to be doing and when, so short, clear instructions are important. These may need to be frequently repeated verbally, or by using visual reminders to help them keep track of what they are being asked to do. Long sequences of instructions, or tasks with multiple steps told to the child in one go are likely to cause frustration and contribute to the child becoming disengaged.

Many of those I spoke with reported successful learning and engagement when things are delivered in smaller chunks, or learning is heavily scaffolded. Again, this appears to relate to the child knowing what is expected of them, and using frequent verbal or visual reminders to keep track of these expectations. It is not unusual for a child with ADHD to frequently forget what they should be doing, however they may also become frustrated if this is just assumed!

Lists **-**Task lists are used by parents to help manage bedtime routines and lay out expectations for the child in advance e.g. steps for having a shower, getting ready for bed.

## Balance of adult involvement and supervision

Children with ADHD may behave in ways that seem young for their actual age. For this reason they are usually supervised by adults in many places across the school. However a balance needs to be drawn between having an adult present for support and interaction or guidance, and allowing the child to develop independence and complete activities on their own (which may boost self-esteem).

## Unstructured times

Children with ADHD struggle to cope when they have long periods of time with little structure. In school this may be lunchtime and playtimes, or ‘free play’ time within the classroom. Having some small options to insert structure into these times can be useful, for example activity or puzzle books that the child can have at lunch. Children with ADHD do not like being too constrained, and value feeling like they have freedom of choice. They would rather pick from a variety of options, with options that change frequently, then having one set ‘menu’ of potential activities (note this might be different for those with traits of Autism who may prefer the set menu). An alternate option is to have a set of cards, each with an activity choice and the child can pick a card (possibly at random) to decide which activity to do.

Children with ADHD also like to be involved in clubs and activities. This is an important place for them to learn new skills.

## Written work

Writing and typing can be difficult for a child with ADHD, although this varies a lot. If a child struggles with writing, the option to type notes may be appropriate. However this may not be helpful if they also struggle to type or spell. Solutions that have worked for some include having a scribe to do the writing, an iPad and dictation. Another option is to provide partially-written documents for the child to edit (rather than writing themselves). Tablets were considered helpful for school work.

## Focus

Children with ADHD will not have the same attention span for every different thing they do during the day. Things that are more or less cognitively and emotionally-demanding will affect their attention, as well as how interesting and engaging the child perceives the thing to be. Many people talked of instances or hobbies where children with ADHD were able to ‘hyperfocus’ and spend long periods of time doing one thing.

In the school context, this means that a child with ADHD may have a very varied attention span depending on what they are doing. They may be able to complete maths problems for 45 minutes without a break, but only manage 5 or 10 minutes of a different subject. Making an effort to understand the frequency of breaks needed and the typical time they can pay attention to a given subject means that activities and tasks can be planned appropriately for different subjects and lessons.

## Self-esteem and success (exclusion and isolation)

Children with ADHD, like other children, want to do well at school and be praised for their success. Unfortunately school can be a very negative part of a child’s life if they have ADHD and are not well-understood or supported. If a child with ADHD dislikes school because they have repeated negative experiences there, this can contribute to school refusal, absence and behavioural disruption at school (especially if the child has learned that behavioural disruption leads to them being allowed to go home).

Many children with ADHD experience patterns of school and social exclusion and isolation. These experiences are reported to be very negative, and can contribute to a child’s negative perceptions of school and individual staff. Isolation booths and reset rooms can be very distressing for these children, particularly if they feel trapped or unable to move around.

Children with ADHD are often aware of the negative perceptions of others, and many people told me of instances where peers, school staff and even other parents had victimised the child with ADHD, leading to negative self-perceptions and poor self-esteem as well as a dislike of school. On occasion, school becomes so distressing for children with ADHD that they may self-harm or experience suicidal thoughts.

On the positive side, there were many instances described of how schools can provide a positive environment for the child, and positive associations with school make education easier for all parties involved. The relationship between a child with ADHD and their teacher in Primary school is key, and these relationships seem to flourish when teachers take time to get to know the child ‘beyond’ their ADHD, when they make efforts for the child to feel included in normal school life (for example being a classroom monitor or having another classroom responsibility, being encouraged to take part in clubs and activities). Giving the child small responsibilities to have a reason to ‘big them up’ is seen as useful. Where children and families feel that they are listened to, positive relationships with school can be established and lead to a supported and successful environment for the child.

## Set them up to achieve

Children with ADHD, like other children, need things to feel proud about. People reported designing learning and other activities to ‘set them up to achieve’ rather than being unable to complete the work. There is a balance needed between setting achievable targets and making sure the child feels appropriately challenged: several people talked about the problem of work being too easy to complete (whether or not answers are incorrect!), however others spoke of the issues of not completing work because of not being able to focus for long enough.

Once you establish a history of achievements, drawing the child’s attention to recalling that event, and how they felt then was used as a way to help engage the child’s focus and willingness to try hard and persist with new or difficult tasks. On the flip side, helping the child to see where they have a pattern of responses and explore the reasons for this may also be useful if habits become entrenched.

Rewards that are individually-tailored and related to things the child values and enjoys are considered more valued than generic rewards.

## Other parents and children

Primary school is not just about the school day. Young children often have birthday parties or other occasions where many of the peer group are invited. Children with ADHD may have been invited to a party and subsequently not invited to other parties, or they may be excluded from even receiving an invite. This can make them feel that their classmates do not like them. Some people have told me that when other families and parents are deliberately inclusive, they really appreciate this. Some families join local ADHD support groups, these are often considered a very valuable place for parents to share experiences and feel supported and understood. Parents of children with ADHD may not know if there are any local parent groups.

## Praise

Children with ADHD like to be praised for achievements, and this becomes increasingly important throughout Primary school. Praise from teachers, senior staff and teaching assistants in particular seems to be valued. Praise should be specific to what a child has achieved, and not just tokenistic- for example “You’ve completed 12 of the 15 maths problems in the time we had. That shows that you’ve been focussing really well and you haven’t rushed just so they were all finished, well done Danny”.

One of the reasons that school can be so negative for children with ADHD is that they become more and more aware of others receiving praise for things that they feel they can never attain. For this reason class-level behaviour systems and public recognition of good behaviour, for example those where the students in the class receive points displayed on a notice board, can be very negative for children with ADHD. They may become aware that they have never been ‘student of the week’ or similar, or note that their name is always at the bottom because they have the fewest merit points.

Praise is also important to break cycles relating to the child’s fear of failure. If they have experienced multiple ‘failings’ because of e.g. being unable to complete set work, specific and targeted praise is a helpful motivator to help a child engage with tasks.

## Flexible expectations

Many of those I spoke to talked about the need for flexibility and give-and-take, with many examples of how a task was unsuccessful with the child, but changing the approach to the task or even trying again on a different day allowed for success. Like other people, children with ADHD might be in a different mood from one day to the next, or from morning to afternoon, and their attitude may vary as well. Being aware of this and not forcing the child to do something within a set time frame if they are not in the right mood is perceived as being more useful than causing the child to lose their temper or create a power-struggle between adult and child.

Flexibility with participation options for different activities was helpful, for example being part of an after-school club with the option to participate with the group or choose to do an activity on their own.

A further area where flexibility was perceived to be needed was around eating and snacking. People felt that children with ADHD are burning through their energy resources quickly as they are so active. Fast metabolisms seemed common, and the need and timing of snacks to help the child regulate their mood was seen as important.

Reduced and flexible timetables were reported to be useful accommodations for children with ADHD in some situations, as long as they were not restricting the child’s participation in normal school life, such as being allowed to go outside at playtime. These timetable flexibilities have been used to address problems with arriving and leaving school (often very busy times in the school day when a lot of people are in one area with little structure) through changing drop-off and pick-up times. Others told me that a child was allowed to come in to school early, and was given a responsibility as ‘register monitor’ to validate why they experienced different treatment to the other students (who were not allowed in until the designated time). This, and other strategies where aversive situations are circumvented or avoided, seem to be successful as long as the child does not feel singled out.

## Group work

Group work is often a challenge for children with ADHD. Those I spoke with said a small group with a maximum of three people was the most conducive way to including children with ADHD in group work. Peers and other pupils can also be a good influence, and children with ADHD, like any other child, may be motivated by being able to work in a group with the ‘cool kid’ or the ‘pretty girl’ (sic). Others feel more at home with other students who are perceived as misfits, or the weird ones. This will obviously vary between children. Where people had experience of different schools, they mentioned the benefits of smaller class sizes.

## Communication and Tensions between school and home

It is sadly normal for relationships between parents and schools to be challenging when a child has ADHD. School staff, including teachers, teaching assistants and mealtime assistants, are often thought to be lacking specific knowledge about ADHD. Parents may try to impart some of what they have learned through the diagnosis and treatment process to school staff with the intention of better supporting the child in school. Some parents said they had attended specific parent training such as 1-2-3 Magic. This could be received poorly by school staff if they considered themselves to already have the required knowledge. Perhaps promoting awareness of the content of parent training will support teachers to understand and implement strategies that parents have found successful.

A few parents mentioned that they had attended school or school trips to support their child. This might be through volunteering as a classroom assistant, or specifically to appease the school where issues had arising and they wanted to find a way to keep the child attending. Sometimes this involved sitting with the child in isolation.

Schools may be judgemental of parenting and even sceptical of the validity of an ADHD diagnosis. People felt that schools should always be accepting of a diagnosis. Although it seems fair to say that a child with ADHD living in a chaotic home environment and getting little sleep may have more issues than ‘just ADHD’, the reality is that often parents are doing their very best to parent a child who does not behave in the way we perceive to be normal for their age, and who won’t sleep until very late at night or only sleep for 2-3 hours in spite of their best efforts (sleep problems are very common for people with ADHD). In addition, given that ADHD has genetic influences, school staff should bear in mind that parents of a child with ADHD may have ADHD or similar traits themselves, which can contribute to a home environment perceived by others to be ‘chaotic’. Given that ADHD was poorly recognised until recently, parents of children with ADHD may have never had the support they needed when they were at school, and because of this may also have negative expectations of schools in general.

People said that systems for consistent and regular communication between teacher and parents were helpful, such as a daily report card or using Apps such as classroom dojo. These needed to be used to communicate positive messages to be considered helpful. Some Apps allow teachers to send a ‘thumbs up’ to parents, but people felt that modes where some individual detail could be provided were most helpful (ties in with importance of specific praise).

## School-level policies

Schools having an inclusion policy was seen to be important, as was the involvement of Educational Psychologists and other professionals (usually associated with CAMHS). Policy should be developed addressing relying on parent involvement to support the child at school during the school day. Events such as Neurodiversity Celebration week can be engaged with by schools. Celebration of individual differences is valued. There should also be policy around reporting and acting on diagnoses, including how to support parents with the Education Health and Care Plan process. Some parents reported that schools had dissuaded them from trying to navigate the process.

Most schools have a code of behaviour with several rules that students are expected to abide by. Children with ADHD struggle to understand behaviour rules that are ambiguous, such as “be ready” or “be safe”. Similarly, school policies with ‘one size fits all’ consequences were not seen as ideal.

Exclusion and isolation were largely seen as ineffective and negative consequences for a child with ADHD, but were not uncommon. In the event that a child with ADHD is excluded, schools should develop re-integration guidelines to help support the transition back in to school.

Consistency between school staff was considered important to support a child with ADHD to do well in Primary school, this referred to teaching assistants and teachers. This was an issue where children had established a strong bond with their teacher, only to have a different teacher the following term. In a similar vein, children with ADHD often find transition difficult. It is important to plan for and support transitions for children with ADHD in school, whether this is transition from one school to another, or transition between teachers and classrooms.

## Fitting in to school

Children with ADHD seem to be happiest when there is a good ‘fit’ between the school ethos and environment and their personality. The school ethos is perceived to be set by the head teacher, and school environment and culture vary widely from school to school. People felt it would be helpful if schools could work to show their staff had an understanding of ADHD, and communicate this knowledge to parents. One important component of this is understanding the relationship between ADHD and sleep (children with ADHD often do not sleep many hours at all).

Some people have told me of schools that refuse to ‘acknowledge’ ADHD as a clinical construct. This is very difficult for parents who have been told by specialist healthcare staff that their child has a medical diagnosis, and the school refuses to accept it. Schools that have an ethos of nurture, play and a focus on happiness and wellbeing seem to be those where children with ADHD are most comfortable.

On the other hand, some parents may not understand or appreciate the lengths that a school, SENCo or teacher are going to in order to support their child. This is likely to be due to poor communication from school to home. Often it seems the only time that schools and parents are in contact is for negative situations or behaviour incidents. Opening up channels for positive communication between parents and school is important.

## Less waiting

Many children with ADHD will find waiting or delays unbearably frustrating. Having smaller lines when students are asked to line up, and finding other ways to reduce unstructured ‘waiting’ time seems to be useful.

## Do things gradually

If trying to engage a child with ADHD in something they do not like or a difficult topic, ease them in to it by starting with a comfortable conversation or activity. For example, if a child has decided that they hate Mr X and now refuse to attend lessons where he is in the room, it may be very difficult to understand what happened that made them feel that way towards Mr X. Initiating a conversation about food, animals or games (comfortable territory) is a useful tool to begin to broach more difficult subjects. For example, you might discuss with the child what they like to play with at home, then what they play with together with their siblings. You might then introduce the idea that sometimes siblings have arguments or you might feel angry towards them sometimes, discuss around this, then transfer to discussing why they might have felt that way towards Mr X and exploring that situation.

## Active listening and building empathy

Children with ADHD often feel ignored or misunderstood. Simply feeling that they have been listened to is appreciated and reportedly impacts on how they feel about school or a teacher. Active listening, where you listen to what the child says and then reflect it back will show the child that you have heard them (e.g. “it sounds like you are saying that you were having a great time playing football with Jenny, but then something made you angry. Is that right?” etc).

Related to active listening, people said how important it was to build an empathic relationship with the child, which involves patience and showing empathy for the child’s emotions. Many children with ADHD feel misunderstood. Self-esteem can be boosted by feeling that they have someone (who the child respects) who listens to them.

## Support self-regulation

Many of the things that people said works for ADHD seem to come down to supporting the child to regulate their own behaviour, cognitive engagement and emotions and therefore manage their ADHD symptoms. Relaxation strategies, physical activity to promote core strength, opportunities to move around freely or ‘fidget’ using a moving cushion or fidget spinner were seen as useful. What is useful for one child may not be useful for another, for example fidget spinners were a distraction for one child rather than being helpful for regulation.

Breaks are seen to be important, alongside the option to withdraw to a safe space. These include provision for a child to remove themselves from a group situation if need be (having a card they can hand to the teacher which allows them to leave to a designated quiet space). Allowing empty time, brain breaks and sensory breaks were mentioned by many people that I spoke to. It was seen to be helpful for the child to be able to access areas of the school outside of the classroom for quiet space or breakout zones. Executive functions are another area where people thought support was needed.

## How do you get to know a child with ADHD?

This relates specifically to the child strengths part of the toolkit (know the child), and what child-led activities would be appropriate for getting to know more about individual likes and strengths. Different methods of finding out information about the child will be needed depending on the individual and their preferences for this activity. The medium they choose will also tell you something about them! Suggestions include making a “news story” on camera about yourself, creating a visual dictionary of photos that mean something to them that they can then tell you about, creating or completing comic strips, making a film or video, and making a Wikipedia entry.

## Persistence

Many school staff and parents working with children with ADHD talked about the vast array of strategies they have tried. If a strategy does work, do not stop using it! If a strategy doesn’t work for a specific problem, be open-minded to trying it again. Educational psychologists and SENCOs emphasised the need for keeping up with strategies and reinforcements over time, and the importance of trying things again even if they didn’t work the first time- children change, problems change and ‘what might work’ could also change.

## Reflecting on past situations

People have used visual tools to help a child with ADHD understand and reflect on recent problematic situations, for example re-playing the situation before an argument erupted. Using a step-by-step visual approach to map out the child’s understanding of the situation and why they behaved as they did can assist the child to reflect on the situation. Then you can work with them to plan what they might try to do next time if a similar situation arises. Bearing in mind the need for short chunks of instructions, this plan might be a 3-step series of actions that link to more support. For example, 1- *notice I am getting upset*, 2- *notice that this is because of other people*, 3- *go to Mrs Y and tell her I am getting upset*. Mrs Y could then hold and pass on the next part of the plan, for example praising the child for seeking her support rather than starting a fight, visual prompts about how to calm breathing, and steps for resolving the issue and returning to the class.

Children with ADHD can struggle to get past upsetting situations, which can impact on the rest of the school day or week. Some people said they use mindfulness strategies to help the child be ‘in the moment’ rather than ruminating on past situations. Mindfulness involves tuning awareness into the present moment, and the sights and sounds in that moment.

## Keeping occupied

Some people said they use distraction techniques if a child gets fixated on something or is feeling negatively towards an activity. Alongside this, people noted that often when children with ADHD are disruptive, it is because they are not engaged in a meaningful task. Having several options for keeping the child occupied is seen as useful (e.g. different worksheet, small classroom jobs to do).

## Other ADHD treatments (medication)

Some children with ADHD take short-acting medication that wears off quickly, and requires a second dose to be taken during the school day. Others take medication that lasts for the whole day. Making sure there are systems in place for children to access this at school was seen as important. Medication can have large positive impacts for some children with ADHD, someone said it was ‘transformative’ in terms of improving relationships with teachers and peers. There were some negative issues that arose with medication however, such as feeling effects as it wears off, interfering with sleep or appetite, or making fixation/repetitive thinking patterns worse.

## Want to share this document or know more?

Contact:

Dr Abigail (Abby) Russell

Senior Lecturer in Child and Adolescent Mental Health

Children and Young People’s Mental Health Research Collaboration (ChYMe)

University of Exeter Medical School

01392722985 [a.e.russell@exeter.ac.uk](mailto:a.e.russell@exeter.ac.uk)

*You may share this document with individuals for personal use. If you wish to post this online or make it more widely available please contact Dr Abby Russell for permission*

*Content under copyright 2022, The University of Exeter*

*All rights reserved. The copyright and intellectual property rights, the associated content, and the material contained herein are owned by or supplied under licence to the University of Exeter. For further information, usage or translation of the copyright, please contact Innovation, Impact and Business:* [IPcommercialisation@exeter.ac.uk](mailto:IPcommercialisation@exeter.ac.uk)*.*

# Appendix S3. Toolkit description

PG members decided that the toolkit should be primarily a digital resource, (<https://www.toolsforschools.info>). The digital content is supplemented with printable PDF resources, that can be digitally or manually personalised. Teachers are primarily responsible for using and delivering the toolkit to the target student, ideally with the support and oversight of the SENCo. Teaching assistants and other adults can also deliver and support delivery. The research team provided ~20 minutes of training to school staff, however the toolkit also has in-built “walkthrough” videos showing how to use and apply the intervention for both school staff and for parents/carers. Flex is designed to be delivered face-to-face to the child in school and mainly within the classroom. All the components can be delivered to an individual, however many are designed to be deliverable to groups of children in order to align with provision within the classroom.

Flex starts with online training for school staff and parents. Children complete an activity to identify strengths or hobbies that could be used to personalise the intervention (“Know Me”- see below). SENCos and teachers complete a functional behaviour assessment for each child to identify two core behaviours that will be the intervention targets. These are operationalised into tangible and achievable goals (e.g. stay in seat for 5 minutes, with the number of minutes increasing as the child meets the target) over the course of two meetings; the second including parents. A digital daily report card (dDRC) is established with these agreed behavioural goals. SENCos and teachers use a graphical map, the “behaviour web”, linking observable behaviours to ADHD-related outcomes to identify two modules for each child. Modules are individual ADHD-related outcomes selected by consensus in a Delphi study (Perry et al., 2021), including sustaining attention, self-esteem and teacher-child conflict. Modules contain a bank of approximately 10 strategies each that can be used at individual or classroom level to target the outcome. PG input emphasised the importance of including printable resources. Selected strategies are then implemented by teachers, teaching assistants (TAs) or SENCos.

# Appendix S4. Logic models of problem, solution and change

See separate PowerPoint file

# Appendix S5. List of performance objectives for teachers, children and parent/carers

Performance Objectives: Teacher

**Necessary environmental (school/class level) conditions**

**Teacher is knowledgeable about ADHD and unique implications for classroom management**

Undertake toolkit training/psychoeducation about ADHD

Express the understanding that parents are not usually the cause of traits relating to ADHD

Be aware of and access resources on ADHD through e.g. charity websites

Discuss with parents their knowledge and training, and appropriate management and strategies for the child

**Teacher offers flexibility and individual adjustment to the child with ADHD consistently**

Understand and appreciate the child’s strengths and interests

Tailor learning materials for the child to these strengths and interests

Proactively explore with SENCo and parents what options are possible

Monitor the child’s progress and adapt materials where they are not appropriate

**Teacher and parents have frequent open communication about the child**

Use a daily report card format or adapt another existing school system

Agree a contact schedule with parents (daily, weekly) and format, review this half-termly

Provide updates to home in accordance with the schedule (regardless of how parents engage)

**Teacher tailors learning difficulty and volume to the child with ADHD**

Review their weekly lesson plans and ensure each activity has variations appropriate for the child with ADHD

Seek advice from the SENCo where modifications are unclear or the teacher is unsure

Monitor how much work the child completes and in what format in order to develop learning that is acceptable/feasible for the child to be able to attain and receive positive feedback

Advocate for the child’s academic potential and seek additional learning support needed to attain this

**Teacher plans and uses small chunks of material with child with ADHD**

Understand the importance of chunking material for students with attention difficulties

Consider when planning lessons where the material can be divided up

Consult with the SENCo or other teachers where this is not clear

Review completed work to assess whether the approach being used is working

Modify if necessary

**Teacher provides alternate formats for work completion/to demonstrate learning e.g. fill in the blanks, using film or video, typing**

Know what resources are available as options for the child

Seek input from parents, OT and SENCo as to best options

Work with the child to understand their preferences and why these are preferred

Be willing to try several options to see what works best in what setting

***Teacher has respect for child’s parents and views them as partners in ADHD management***

Consider the parents as collaborators in managing ADHD traits

Value parents experience in terms of ADHD training, parent training and lived experience at home

Not attribute the child’s behaviour to parenting/chaos at home

Actively seek parents opinions when new challenges arise

Communicate honestly with parents on a frequent basis about the child’s progress

Consult with parents on target and strategy choices

Tell parents what is working at school

**Core toolkit processes (implement and maintain)**

**Teacher is able to choose, implement and review toolkit strategies**

Report that they feel capable and confident when using the toolkit

Be trained in using and interpreting functional behaviour analysis

Be able to operationalise target behaviours with incremental goals as the target is reached

Be able to select strategies to match the target behaviour with support from the SENCo

Have or gather all the resources needed to implement the strategy

Follow implementation instructions

Complete measures

Review and track progress (proactively or set timely reminders for review points)

Modify, sustain or change strategy based on ongoing review/progress tracking

**Teacher is able to identify specific positive behaviours to praise, and uses labelled praise**

Articulate what specific positive praise is, and describe importance of frequent verbal positive feedback to the success of toolkit strategies

Seek to find a reason to offer praise to the child at least once every 15 minutes

Praise the child for their achievements regularly

Be specific with the child about what they are being praised for “I like the way you wrote down the homework yourself without being reminded”

Be consistent with this approach across the school term/year- integrate into teaching practice

**Teacher understands and implements frequent small reinforcements**

Have a list of what will act as positive reinforcements or engaging materials for the child

Produce a menu the child can choose from

Articulate the importance of smaller-sooner rewards in relation to ADHD theory and apply this reasoning to reinforcement/consequences

Be consistent with implementing reinforcement schedules

Set reminders for themselves to ensure this is being followed

**Teacher supports and provides child opportunity for self-regulation**

Work with the SENCo and child to develop self-regulation plans and rules

Identify an area the child can safely use for breaks from class

Monitor the child throughout the day and offer breaks when appropriate

Communicate with parents about strategies that work at home and what is working at school

Use visual aids and prompts consistently with the child while they are learning to self-monitor

**Teacher recognises child’s strengths and positive attributes**

Work with the child to complete the strengths activity in the toolkit

Take time to understand the child’s story and motivations

Articulate their reflections on the child’s school history thus far

Talk positively about the child with the child, their parents, other staff and the child’s peers

Support the child to tell their class about their ADHD (if appropriate)

Use this knowledge of the child to tailor learning activities

**Teacher is able to recall attention or engagement of the child**

Evaluate what current approaches engage the child’s attention

Use these consistently with the child

Seek SENCo advice and consult toolkit strategies for ideas

**Teacher-child relationship processes**

**Teacher has good relationship with child, as reported by teacher and child**

Develop a shared understanding of their relationship with the child, with the child

Work to be consistently positive, flexible and understanding

Seek out advice and support from colleagues after challenging interactions (so that it does not carry over into future interactions with the child)

Engage with the child on a ‘human level’

Understand the child’s triggers, and avoid any where possible (e.g. do not raise voice)

**Teacher is able to de-escalate disruptive or violent outbursts**

Understand a child’s triggers and sensory challenges

Recognise when behaviour is escalating and intervene with opportunity for self-regulation

Communicate to the child that their perspective is important to them, and they want to develop a shared understanding of the situation

**Teacher is able to calmly discuss past situations with child and explore options for future occurrences of the same scenario**

Understand and regulate their own emotional response prior to discussing with the child

Find a calm time to bring up the topic for discussion

Use visual and other prompts to elicit the child’s point of view

Empathise with feelings of remorse or regret

Highlight positive aspects of behaviour

Draw a plan with the child of how they reacted this time and other options for next time

Decide where to store this for easy access in future situations

Child

**CHILD PERFORMANCE OBJECTIVES**

*POs organised broadly into modules*

For all of these, child will receive positive consequence for successful engagement, completion or attempt

**Paying attention and engaging (including on-task behaviour, aspects of working memory)**

***Child will be able to complete classroom tasks appropriate to their ability in a timely manner***

respond to prompt to recognise if they are engaged with what a set task

attempt to reengage following prompt

work on task for NN unsupported

work on task for NN with adult support and encouragement

**Holding back inhibiting, stop and think (targeting hyperactivity/impulsivity)**

***Child will identify impulsive thoughts and behaviour, judge appropriateness in given situation and consciusly try to inhibit using strategies with support***

recognise and articulate impulses

recognise teacher/staff signals that now is a time to resist impulsive behaviour

attempt to reengage following resisting impulse

choose and implement an alternate strategy to use to replace impulse to get up e.g. fiddle toy

**Planning and reminding (organisation skills, some executive functions)**

***Reminding***

***Child will be able to consistently engage with tasks and re-engage after attention has wandered***

recognise articulate the need to be reminded to keep on track

choose method or tool for reminding (such as timer, teacher using certain phrase)

react at prompts from reminding tool in a positive manner and try their best

***Prioritising or juggling tasks***

***Child will be able to identify when they have multiple tasks, prioritise them and attend to these in turn***

identify when they have more than one thing to do with adult support

choose which order to do these in using prompts

assign time allowed for each activity

follow plan and action other needed skills such as reminding (to keep to time)

reassess on completion of prioritised tasks

***Keeping track (Working memory)***

***Child will be supported by adults to keep track of what they need to do in the short term***

use planning skills with adult to make a list of what to keep track of

use reminding skills to check in on that list

identify and successfully reengage with task if they lose track

***Planning***

***Child will express knowledge of what is happening now and next, and plan ahead for those things***

list the items they need for the next activity (with or without adult support, with or without visual aids)

find items on the list

put items in a safe designated place until they are needed

***Organisation***

***Child will follow a strategy for organising school materials***

have designated places for school materials such as tablet, pencils

decide where those designated spaces are in conjunction with teacher/TA

use organisation system

notice if item not in place in designated place

check the context and decide if they should put their hand up before getting out of their seat to locate missing items

check their key locations for item (using key locations prompt to say which places to check)

seek adult help when checked all locations if not found

**Knowing me (the child)**

***Child is able to consciously recognise their emotional and behavioural regulatory state***

Be able to communicate their emotions or levels of arousal through use of a visual tool

Perform a consciously ‘check-in’ and communicate their emotional/regulatory state

Identify and action a visual prompt/reminder of what to do next (e.g. wave yellow card at teacher, take 5 deep breaths)

Child is able to verbalise/signal need to use self-regulation strategy to appropriate adult/peer

Child can access and implement actions prompted by a reminder using visual or verbal tools

Child implements and follow the steps in the reminder in sequence (e.g. raise your yellow card and when the teacher nods then get out your chair and move to the quiet space)

***Child is able to self-regulate/self-monitor and correct through use of toolkit strategy (either on own or with appropriate adult/peer) in the immediate term***

Child articulates the cause of their dysregulation (event or feeling)

Child can judge the relative size of the problem, using non-verbal or verbal signalling and with support

Child decides on appropriate reactions and solutions for problems of different sizes

Child can choose and carry out a solution to the problem that corresponds to it’s size

Child has the materials for strategy (or is given them) or gets them from a known location

Child uses materials/follows steps of strategy

Child reviews their emotional state, continues if necessary or desists if successfully back on track

***Child is able to recognise environmental triggers and their response to them***

Child recognises and remembers what things at school make them more likely to do a ‘behaviour’

Child follows agreed protocol when a trigger is present (e.g. asking if they can move to another group or do a different activity)

Child seeks support from appropriate adult through verbal or non-verbal actions

**Connecting (relationships, conflict, communication)**

***Child has positive relationship with peers***

Articulate the aspects of positive social behaviour

Identify situations where behaviours are and are not appropriate

With support, choose the type of social behaviour to use when in different situations

Child will have a friend with whom they talk and choose to spend time with

***Child has positive relationship with teacher***

Express the belief that the teacher views them positively and understands them

Express positive characteristics about their teacher and how they can support them (the child) in school

Engage positively with teacher requests and comply

Have and use agreed strategies to make the teacher aware they want to communicate with them

(most of this comes from teacher POs)

***Child is able to resolve and move on from conflict situations***

Identify when a social situation is negative using verbal or non-verbal signals

Identify the size of the problem using knowing me skills

Take the appropriate action from the choices for that size of problem, for example removing from the situation, taking 5 deep breaths

Implement self-regulation from knowing me

Talk to the teacher or another adult after the conflict

Reflect on the conflict to make a plan of action for if a similar situation should happen again

Parent/carer

**Parent is actively engaged with school’s approach to support child**

Undertake the toolkit training

Agree the toolkit is appropriate for their child with school staff

Support teachers to understand their child’s strengths, interests, triggers and potential reinforcers (rewards)

Attend initial meetings (via zoom) to understand and support operationalisation of problem behaviours

Agree with teachers on chosen strategies, and implement any home-based component as requested

**Parent feels valued and respected (equal) to school staff**

Express the belief that their thoughts are listened to and acted on by school staff

Report that they have a trusting relationship with the teacher

**Parent communicates with teacher on frequent basis**

Engage with decision about daily report card

Communicate with teacher via this at agreed frequency

When major events happen at home, communicate this to school in agreed manner

Agree with teacher best form of communication and what to communicate about

**Parent shares knowledge of ADHD and strategies that work at home with school**

Express the belief that their knowledge is sought and valued by school staff

Reflect on strategies tried (openly and honestly) with school staff

Understands what a strategy is

**Parent implements and sustains reinforcement of toolkit strategies at home**

Understand how to use the toolkit

Be able to access strategies in use and follow parent instructions on the digital platform

Access and use (or receive from school) needed resources

Discuss any queries with the teacher/SENCo

Follow the strategy rules and schedule

**Parent undertakes learning relating to ADHD and behaviour management**

Undertake the toolkit training for parents

Be able to list other possible sources of support such as ADHD groups and parent training

Act on seeking out other sources of support

Seek further knowledge using their own social networks, the toolkit resources and other relevant sources

***Parent seeks relevant new knowledge about ADHD and its’ management***

Be aware of where ADHD knowledge is published/publicised

Demonstrate how to judge the validity/evidence around new ADHD information

Talk about the role of myths and rumours in common beliefs about ADHD (stigma)

Seek advice from healthcare professionals about their child

# Appendix S6. Screenshots from the Flex toolkit

## Landing page (senior leadership login version shown)

## Know ADHD page

## Know Me page

## Example printable prompts for home for Know Me activity

## Setting Goals and Targets page

## Example meeting template for setting goals and targets

## Behaviour web

## Child toolkit page

## Example module home-page with strategy short descriptions

## Example strategy page

## Examples of PDF resources to accompany strategy delivery

# Appendix S7. Planning group input on involvement of child in Flex and home interaction with toolkit

### Child awareness of intervention

PG members emphasised that different families would feel differently about their child being aware and involved in discussions about the toolkit. Some were very clear they would like their child/themselves to be aware and involved as much as possible. Others were very clear that they would not want their child to know about the toolkit being used explicitly, or attend meetings about the toolkit. This was usually because it was considered inappropriate for the child’s age or developmental stage, or because they felt their child would consider that this meant there was something wrong or different about them compared with other students, and did not want to reinforce this. Therefore, the informed consent process made it optional for families to involve their child: at one extreme, they could choose to tell their child nothing and consent on their behalf. At the other end of the scale, children could read and watch appropriate information sheets about the research study, meet with the researcher doing the consent visit, and add their signature to their consent form.

### Home interaction with toolkit

Although initially proposed as a school-based intervention, PG members felt that involvement of home in supporting this was critical. They discussed the range of potential engagement required for Flex to be useful, and it was felt that Flex would need to be able to function across the breadth of no home involvement, to intensive engagement. There was also discussion around which features of the website interface home could be allowed to view, to edit, or to add content to, taking into account the power dynamic and often-negative home-school relationship patterns that develop for families of children with ADHD. The resulting prototype had a home login, where the user could access the Know ADHD videos, access and add to the Know Me activity (including resources and worksheets to do this activity at home) as well as see what the teacher had entered into this section. They could view but not edit targets entered by the teacher, strategies activated, and graphs showing behaviour over time (captured from the repeat quantitative measures in the research project). Home could add and reply to the Daily Report Card.

# Appendix S8. Key recommendations for co-design of behaviour change interventions (summary of those covered in the main paper discussion)

1. Ensure you choose an intervention development method that provides structure for the overall intervention development process that can be conveyed clearly to members of the public
2. Engage a diverse group of people to contribute to co-design, and be flexible to accommodate continued enthusiastic input as well as engaging with new perspectives or specific public groups where needed to bolster your ability to make informed decisions
3. Give co-design members choice over whether and how their information is shared more widely within the group
4. Be prepared to run the same activity on multiple occasions and in multiple formats so that you can gather all the relevant views
5. Be open and honest where there are different opinions, but have clear communication about who will make final decisions. Take these back to the group with an explanation of why this was the chosen way forward
6. Acknowledge potential for power imbalances and ensure all opinions can be shared and are shown to be valued
7. Where there are resource or project constraints, use co-production where is can be most influential for the project
8. Do not underestimate tasks that can be co-produced, break these down into smaller activities so that they do not become overwhelming for non-academics.
